# Severe *Strongyloides stercoralis* infection in kidney transplant recipients: A multicenter case-control study

**DOI:** 10.1371/journal.pntd.0007998

**Published:** 2020-01-31

**Authors:** Lísia Miglioli-Galvão, José Osmar Medina Pestana, Guilherme Lopes-Santoro, Renato Torres Gonçalves, Lúcio R. Requião Moura, Álvaro Pacheco Silva, Lígia Camera Pierrotti, Elias David Neto, Evelyne Santana Girão, Cláudia Maria Costa de Oliveira, Cely Saad Abboud, João Ítalo Dias França, Carolina Devite Bittante, Luci Corrêa, Luís Fernando Aranha Camargo

**Affiliations:** 1 Infectious Diseases Unit, Universidade Federal de São Paulo, São Paulo, Brazil; 2 Kidney Transplant Unit, Hospital do Rim and Universidade Federal São Paulo, São Paulo, Brazil; 3 Department of Preventive Medicine, Universidade Federal do Rio de Janeiro, Rio de Janeiro, Brazil; 4 Kidney Unit, Universidade Federal do Rio de Janeiro, Rio de Janeiro, Brazil; 5 Kidney Transplant Unit, Hospital Israelita Albert Einstein, São Paulo, Brazil; 6 Kidney Unit, Universidade Federal de São Paulo,São Paulo, Brazil; 7 Kidney Transplant Unit, Universidade de São Paulo, São Paulo, Brazil; 8 Kidney Transplant Unit, Universidade Federal do Ceará, Ceará, Brazil; 9 Infectious Diseases Unit, Instituto Dante Pazzanese de Cardiologia, São Paulo, Brazil; 10 Department of Epidemiology and Statistics, Instituto Dante Pazzanese de Cardiologia, São Paulo, Brazil; 11 Infectious Diseases Unit, Hospital Israelita Albert Einstein, São Paulo, Brazil; PUCRS, BRAZIL

## Abstract

**Background:**

Severe *Strongyloides stercoralis* infection in kidney transplant recipients is associated with considerable morbidity and mortality, although little is known about the risk factors for such infection.

**Methodology/Principal findings:**

This was a retrospective, multicenter, case–control study in which we assessed the risk factors for and clinical outcomes of severe *S*. *stercoralis* infections in kidney transplant recipients in Brazil. We included 138 kidney transplant recipients: 46 cases and 92 controls. Among the cases, the median number of days from transplantation to diagnosis was 117 (interquartile range [IQR], 73.5–965) and the most common clinical findings were gastrointestinal symptoms (in 78.3%) and respiratory symptoms (in 39.1%), whereas fever and eosinophilia were seen in only 32.6% and 43.5%, respectively. The 30-day all-cause mortality among the cases was 28.3% overall and was significantly higher among the cases of infection occurring within the first three months after transplantation (47% *vs*. 17.2%, *P* = 0.04). The independent risk factors were receiving a transplant from a deceased donor (odds ratio [OR] = 6.16, 95% confidence interval [CI] = 2.05–18.5), a history of bacterial infection (OR = 3.04, 95% CI = 1.2–7.5), and a cumulative corticosteroid dose (OR = 1.005, 95% CI = 1.001–1.009). The independent predictors of mortality were respiratory failure (OR = 98.33, 95% CI = 4.46–2169.77) and concomitant bacteremia (OR = 413.00, 95% CI = 4.83–35316.61).

**Conclusions/Significance:**

Severe *S*. *stercoralis* infections are associated with considerable morbidity and mortality after kidney transplantation. In endemic areas, such infection may occur late after transplantation, although it seems to be more severe when it occurs earlier after transplantation. Specific risk factors and clinical manifestations can identify patients at risk, who should receive prophylaxis or early treatment.

## Author summary

*Strongyloides stercoralis* is a soil-transmitted helminth found in tropical and subtropical regions worldwide. Although infections are usually mild or asymptomatic, they can be severe, with high mortality rates, in patients receiving immunosuppressive therapy, including organ transplant recipients. It is therefore relevant for countries endemic for infectious diseases such as strongyloidiasis to have a special focus on endemic diseases in organ transplant recipients. In this study, we describe the risk factors for and clinical findings of severe infection with *S*. *stercoralis* in kidney transplant recipients in Brazil. We retrospectively collected data from the medical charts of kidney transplant recipients diagnosed with severe strongyloidiasis, comparing them with those obtained for a control group of kidney transplant recipients with similar characteristics and no signs of strongyloidiasis. Gastrointestinal and respiratory symptoms were common, and 30-day crude mortality was 28.3% among the infected patients. Mortality was significantly higher when infection occurred within the first three months after transplantation, and we identified specific risk factors for *S*. *stercoralis* infection. Because there is little available information about severe strongyloidiasis in kidney transplant recipients, we believe that specific risk factors and clinical manifestations could identify patients at risk, who should receive prophylaxis or early treatment.

## Introduction

Infections due to the nematode *Strongyloides stercoralis* are endemic in many countries, and the most recent data suggest more than 300,000,000 patients are affected worldwide [1]. In Brazil, such infections are identified in 5.5–29.2% of the patients screened, depending on the diagnostic methods employed [2].

Although the great majority of patients infected with *S*. *stercoralis* are asymptomatic or have only a few, mild clinical symptoms, a small proportion of such patients present with severe disease, manifesting either as hyperinfection syndrome or as disseminated disease [3,4]. Such infections mainly affect immunocompromised patients such as those on long-term corticosteroid therapy, those with AIDS, and organ transplant recipients [5,6]. Severe gastrointestinal and respiratory symptoms are the main clinical manifestations, and the diagnosis is typically delayed, resulting in high mortality [7,8].

Kidney transplant recipients are at risk for severe *S*. *stercoralis* infection because of the intense immunosuppression to which they are subjected. Donor-transmitted strongyloidiasis has been well documented, and case series have reported mortality rates as high as 68% in hyperinfection syndrome or disseminated disease [9–12]. However, because the data are generated mainly from case reports or single-center case series, the real impact of severe *S*. *stercoralis* infection is poorly understood, as are the risk factors for such infection and the associated mortality.

The objective of the present study was to assess the clinical impact of severe *S*. *stercoralis* infection in kidney transplant recipients in Brazil, as well as to identify the risk factors and prognostic factors for such infection.

## Methods

### Ethics

The study was originally approved by the Research Ethics Committee of the Federal University of São Paulo, in the city of São Paulo, Brazil (Reference no. 1.009.570), subsequently being approved by those of the other participating institutions. Because it was an observational retrospective study, the requirement for informed consent was waived. All of the researchers signed a data use agreement protecting the confidentiality of the data.

### Study population and study design

This was a retrospective case–control study conducted at six Brazilian kidney transplant centers located in three different Brazilian states: two in the southeastern region of the country (São Paulo and Rio de Janeiro) and one in the northeastern region (Ceará). Data were collected from 1999 to 2015. The case–control ratio was 1:2.

Cases were included on the basis of the following criteria: being a kidney or kidney–pancreas transplant recipient; being ≥ 18 years of age; and having been diagnosed with severe *S*. *stercoralis* infection—characterized by severe symptoms attributed exclusively to the *S*. *stercoralis* life cycle (intense respiratory, gastrointestinal, or other symptoms), together with the isolation of *S*. *stercoralis* in sputum, tracheal aspirates, bronchoalveolar lavage fluid, duodenal aspirate, stool samples, pulmonary biopsy samples, or gastrointestinal biopsy samples. None of the patients included showed evidence of larvae in organs other than intestine and lungs. Due to the retrospective nature of this study, we were not able to determine the occurrence of heterogeneous infection. Patients complaining of mild diarrhea or upper gastrointestinal symptoms were excluded if those symptoms were managed at outpatient facilities.

The selection criteria for the matching controls were as follows: having undergone transplantation at the same center and ± 40 days of the same date as the respective case; being of an age ± 20 years of that of the respective case; having a functioning graft at the time of diagnosis of the respective case; showing no clinical or biochemical evidence of severe *S*. *stercoralis* infection after transplantation.

Cases were identified from discharge diagnosis codes and from the results of laboratory tests for *S*. *stercoralis*. Transplant centers in Brazil do not routinely screen for *S*. *stercoralis* infection before transplantation. During the study period, none of the participating centers performed systematic surveillance for *S*. *stercoralis* infection after transplantation and severe infections were managed at the discretion of the physicians involved.

### Data collection

Data were collected directly from patient charts, as well as from the laboratory records, at each center. Cytomegalovirus (CMV) infection was defined as outlined in the international consensus guidelines on the management of CMV in solid-organ transplantation [13]. Previous viral, bacterial, mycobacterial, parasitic, and fungal infections were defined on the basis of clinical aspects and culture results (isolation of the agents from sterile sites) in medical charts and of the treatment instituted by the attending physicians. Such infections were categorized as previous infections if they were diagnosed within the six months preceding the diagnosis of severe *S*. *stercoralis* infection. Coinfection was defined as the occurrence of another infection (characterized as above) immediately after the diagnosis of severe *S*. *stercoralis* infection. Graft loss was defined as the need for chronic dialysis or surgical graft removal. Rejection treatment was categorized as empirical or as histologically defined. Eosinophilia was defined as > 450 cells/μl at the time of diagnosis of severe *S*. *stercoralis* infection [14]. Prophylaxis against *S*. *stercoralis* infection was defined as the use of any anthelmintic, such as ivermectin, albendazole, or thiabendazole, at or immediately after transplantation. The cumulative corticosteroid dose was defined as the sum of daily corticosteroid dose administered from transplantation until the date of diagnosis of severe *S*. *stercoralis* infection per mean patient weight during the same period (mg/kg of prednisone). Significant graft dysfunction was defined as an at least two-fold increase in serum creatinine over the pre-admission levels (mean value of the last three measures). Delayed graft function was defined as the need for dialysis for up to 7 days after transplantation. Early and late infection were defined as severe *S*. *stercoralis* infection occurring within the first 90 days after transplantation and thereafter, respectively. The crude mortality rate was defined as all-cause mortality within the first 30 days after diagnosis of severe *S*. *stercoralis* infection. Respiratory failure was defined as the need for mechanical ventilation following the diagnosis of severe *S*. *stercoralis* infection. Induction therapy was defined as the use of thymoglobulin immediately after transplantation.

### Statistical analysis

Continuous variables are presented as mean and ± standard deviation or as median and interquartile range and were compared by the Mann–Whitney U test. Categorical variables, which are presented as absolute and relative frequencies, were compared by Fisher’s exact test. To analyze risk factors for severe *S*. *stercoralis* infection and for 30-day mortality, we performed backward stepwise multivariate logistic regression. Variables with a *P* value < 0.10 in the univariate analysis were included in the first step of the multivariate analysis. Because the cumulative corticosteroid dose is a relevant variable, regarded as an independent risk factor in other studies, it was included in the multivariate model irrespective of its *P* value in the univariate analysis. We calculated odds ratios (ORs) and the respective 95% confidence intervals (CIs). Patient survival was estimated by the Kaplan–Meier method, and the log-rank test was used in order to compare differences in survival. All statistical analyses were performed using the IBM SPSS Statistics software package, version 19.0 (IBM Corporation, Armonk, NY, USA) and the program R, version 3.6.1 (R Development Core Team—www.r-project.org). Values of *P* ≤ 0.05 were considered statistically significant.

## Results

During the study period, 15,860 transplants were performed and a total of 53 patients were diagnosed with *S*. *stercoralis* infection. Of those 53 patients, 46 (45 recipients of a kidney transplant and one recipient of a kidney–pancreas transplant) met the criteria for severe *S*. *stercoralis* infection (an incidence of 0.29%) and were matched to 92 control patients. The main clinical and epidemiological features of the 46 cases are shown in Table 1. Of those 46 patients, 36 (78.3%) had gastrointestinal symptoms, 18 (39.1%) had respiratory symptoms, and 20 (43.5%) had both. Notably, most of the patients complained of epigastric pain, which could reflect a high burden of parasites damaging the intestinal mucosa or could be a clue for the differential diagnosis with other intestinal complications after transplantation. Only 15 (32.6%) of the patients had fever, and 20 (43.5%) had eosinophilia. Among the patients with eosinophilia, the median eosinophil count was 1304 cells/μl. We found it surprising that the patients with eosinophilia had a median cumulative corticosteroid dose of 93.7 mg/kg, compared with 58.2 mg/kg for those without eosinophilia (*P* = 0.1). The typical periumbilical lesions, a possible key feature for a diagnosis of *S*. *stercoralis* infection, were found infrequently in our sample.

**Table 1:** Clinical and biochemical data related to 46 kidney transplant recipients with severe *Strongyloides stercoralis* infection.

Characteristic	Cases (n = 46)
**Symptoms, n (%)**	
Gastrointestinal	
Epigastric pain	36 (78.3)
Vomiting	29 (63)
Nausea	24 (52.2)
Loss of appetite	21 (45.7)
Diarrhea	21 (45.7)
Weight loss	18 (39.1)
Dyspepsia	16 (34.8)
Constipation	3 (6.5)
Abdominal distension	2 (4.3)
Respiratory	
Dyspnea	18 (39.1)
Cough	8 (17.4)
Hemoptysis	4 (8.7)
Bronchospasm	1 (2.2)
**Skin lesions, n (%)**	5 (10.9)
**Fever, n (%)**	15 (32.6)
**Diagnosis, n (%)**	
Eosinophilia	20 (43.5)
***Strongyloides stercoralis* detection, n (%)**	
Gastric or duodenal biopsy	18 (39.1)
Feces	16 (34.8)
Respiratory samples	6 (13.1)
Colonic biopsy	2 (4.3)
**Treatment, n (%)**	43 (93.5)
Ivermectin	35 (81.4)
Combination therapy	25 (58.1)
**Duration of treatment (days), median (IQR)**	11 (7–20)
**Hospital stay (days), median (IQR)**	29.5 (10.7–47.7)

IQR: interquartile range.

Prophylaxis against *S*. *stercoralis* infection was employed in 28 (60.9%) and 37 (40.2%) of the cases and controls, respectively (*P* = 0.8). None of the patients received prophylaxis with ivermectin; prophylaxis with albendazole was administered in 26 (56.5%) of the cases and 34 (37.0%) of the controls; and prophylaxis with thiabendazole was administered in two (4.3%) of the cases and three (3.3%) of the controls. In all of the patients, prophylaxis was administered at the time of transplantation. Albendazole was administered at 400–800 mg/day for 1–7 days (typically 400 mg/day for 5 days), and thiabendazole was administered at 1000 mg/day for 3–10 days (typically 1000 mg/day for 3 days). Among the cases, the use of prophylaxis against *S*. *stercoralis* infection was associated with a delayed occurrence of infection (182 days *vs*. 87.5 days) and with a lower mortality rate (14% *vs*. 50%), as can be seen in Table 2.

**Table 2:** Comparison and univariate analysis of the cases receiving and not receiving prophylaxis against *S*. *stercoralis* infection, by clinical variables.

Variable	Prophylaxis		OR (95% CI)	*P*
	Yes	No		
	(n = 28)	(n = 18)		
Days from transplantation to infection, median (IQR)	182 (85–1492)	87.5 (62–173)		0.06
Deceased donor, n (%)	24 (85.7)	16 (88.9)	0.75 (0.12–4.59)	1.00
Cumulative corticosteroid dose (mg/kg), median (IQR)	73.6 (41.7–203.0)	67.5 (27.7–117.2)		0.51
Death, n (%)	4 (14.3)	9 (50.0)	0.17 (0.04–0.68)	0.02
Cytomegalovirus coinfection, n (%)	9 (32.1)	1 (5.6)	8.05 (0.92–70.3)	0.06
Previous bacterial infection, n (%)	13 (46.4)	9 (50.0)	0.87 (0.27–2.84)	1.00
Septic shock, n (%)	7 (25.0)	3 (16.7)	1.67 (0.37–7.52)	0.72
Gram-negative bacteremia, n (%)	0	4 (22.2)		0.02
Respiratory failure, n (%)	8 (28.6)	5 (27.8)	1.04 (0.28–3.88)	1.00

CI: confidence interval.

**Fig 1:**
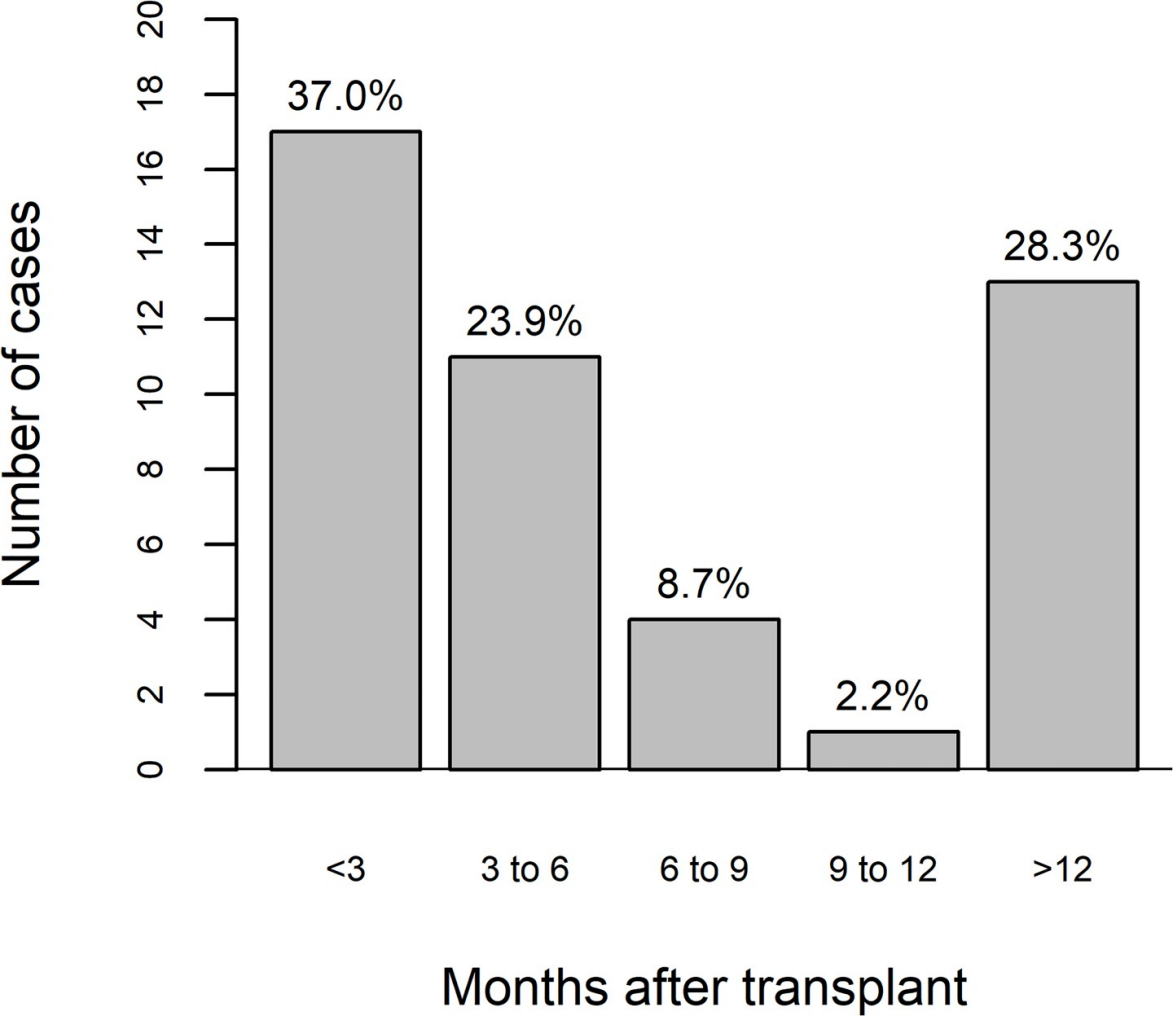
Temporal distribution of severe *Strongyloides stercoralis* infection after kidney transplantation.

The median time from transplantation to the diagnosis of *S*. *stercoralis* infection was 117 days (interquartile range, 73.5–965), 28 (60.9%) of the cases being diagnosed in the first 6 months after transplantation, five (10.9%) being diagnosed between 6 months and one year after transplantation, and 13 (28.3%) being diagnosed thereafter (Fig 1).

Treatment for infection with *S*. *stercoralis* was administered in 43 of the 46 cases; in the three remaining cases, the diagnosis was made *post mortem*. In contrast with the choice of drugs used for prophylaxis, ivermectin, either as monotherapy or in combination with other drugs, was used for the treatment of severe infection in more than 80% of the cases. It is noteworthy that nearly 60% of the patients diagnosed with severe infection with *S*. *stercoralis* were treated with a combination of drugs, typically ivermectin and albendazole.

*Strongyloides stercoralis* was isolated mainly from gastrointestinal specimens. Diagnostic techniques for identifying parasites in stool specimens were employed in 16 (34.8%) of the 46 cases: the Hoffman–Pons–Janer technique was employed at three centers; the Baermann concentration technique was employed at one center; and Rugai method was employed at two centers. In most cases, it was necessary to use an invasive method to diagnose *S*. *stercoralis* infection, probably reflecting a low rate of suspicion or the poor diagnostic yield of stool cultures. Gastric or duodenal biopsy samples were used in 18 (39.1%) of the cases, and colonic biopsy samples were used in two (4.3%). Pulmonary samples, such as sputum, bronchoalveolar lavage fluid and tracheal aspirate, were used in six cases (13.1%).

Table 3 summarizes the clinical complications associated with severe *S*. *stercoralis* infection. Respiratory failure occurred in 13 (28.3%) of the cases; septic shock (which could also be considered a consequence of disease progression and worsening) occurred in ten (21.7%); and infection with gram-negative bacilli occurred in five (10.9%), resulting in bacteremia in four and meningitis in one. The 30-day all-cause mortality rate was high (28.3%). Notably, coinfection with CMV occurred in 10 cases (21.7%). When censored for death, definitive graft loss did not occur in any of the cases, although significant graft dysfunction occurred in 15.2%.

**Table 3:** Complications and clinical outcomes of severe *Strongyloides stercoralis* infection in kidney transplant recipients.

Events	Cases
	(N = 46)
	n (%)
**Complications**	
Respiratory failure	13 (28.3)
Septic shock	10 (21.7)
Gram-negative bacillary infections	
Bacteremia	4 (8.7)
Meningitis	1 (2.2)
Gastrointestinal bleeding	2 (4.3)
Paralytic ileus	2 (4.3)
Malabsorption	1 (2.2)
**Outcome**	
Death	13 (28.3)
Significant graft dysfunction	7 (15.2)
**Coinfection**	12 (26.1)
Cytomegalovirus	10 (21.7)
Oral or esophageal candidiasis	4 (8.7)
Herpetic esophagitis	1 (2.2)
Pulmonary tuberculosis	1 (2.2)

To determine whether *S*. *stercoralis* infection occurring early after transplantation has a different clinical course than does that occurring later, we compared cases occurring before and after post-transplantation month 3 (Table 4). Although the groups were comparable in terms of the major epidemiologic features (including donor features, use of induction therapy, treatment of rejection, and delayed graft function), respiratory failure was significantly more common in early infection than in late infection, as was 30-day all-cause mortality.

**Table 4:** Comparison and univariate analysis of early and late severe *Strongyloides stercoralis* infection, by clinical and demographic characteristics.

Characteristic	Early infection	Late infection	Univariate analysis	
	(n = 17)	(n = 29)	OR (95% CI)	*P*
**Age (years), mean (±SD)**	49.3 (±13.6)	48 (±14.1)	0.99 (0.95–1.04)	0.76
**Male, n (%)**	10 (58.8)	18 (62.1)	1.15 (0.34–3.89)	1.00
**Race, n (%)**				
White	8 (50.0)^1^	16 (55.2)	-	0.19
Mixed	2 (12.5)^1^	8 (27.6)	-	
Black	6 (37.5)^1^	5 (17.2)	-	
**Months on dialysis before transplantation, median (IQR)**	39 (36–60)	48 (17–91)	1 (0.98–1.02)	0.76
**Days from admission to diagnosis, median (IQR)**	5 (3–11)	6 (2–11)	1.005 (0.96–1.05)	0.87
**Deceased donor, n (%)**	16 (94.1)	24 (82.8)	0.3 (0.03–2.81)	0.39
**Induction therapy, n (%)**	12 (70.6)	17 (58.6)	0.59 (0.16–2.12)	0.53
**Delayed graft function, n (%)**	12 (70.6)	18 (62.1)	0.68 (0.19–2.46)	0.75
**Graft rejection, n (%)**	5 (29.4)	8 (27.6)	0.91 (0.24–3.43)	1.00
**Prophylaxis, n (%)**	8 (47.0)	20 (69.0)	2.5 (0.73–8.6)	0.21
**Complications, n (%)**				
Respiratory failure	8 (47.0)	5 (17.2)	0.23 (0.06–0.91)	0.04
Septic shock	6 (35.3)	4 (13.8)	0.29 (0.07–1.25)	0.14
Gram-negative bacteremia	2 (11.8)	2 (6.9)	0.56 (0.07–4.35)	0.62
**Outcome, n (%)**				
Death	8 (47.0)	5 (17.2)	0.23 (0.06–0.91)	0.04
Significant graft dysfunction	2 (11.8)	5 (17.2)	1.56 (0.27–9.1)	1.00
**Coinfection, n (%)**				
Cytomegalovirus	6 (35.3)	4 (13.8)	0.29 (0.07–1.25)	0.14
Oral or esophageal candidiasis	3 (17.6)	1 (3.4)	0.17 (0.02–1.75)	0.14
Herpetic esophagitis	1 (5.9)	0	–	0.37
Pulmonary tuberculosis	0	1 (3.4)	–	1

^1^ n = 16.

The univariate analysis of risk factors for severe *S*. *stercoralis* infection is summarized in Table 5. In the multivariate analysis, receiving a graft from a deceased donor, a cumulative dose of corticosteroids, and previous bacterial infection were independently associated with the development of severe *S*. *stercoralis* infection. In our sample, there were 44 previous bacterial infections that had been treated with antibiotics, all occurring at least six months before the diagnosis of severe *S*. *stercoralis* infection Of those, 28 (63.7%) were urinary tract infections, nine (20.5%) were respiratory infections, five (11.3%) were infections of the skin or soft tissue, and two (4.5%) were bacteremia. The use of cyclosporine and a previous fungal infection were marginally significant (Table 5).

**Table 5:** Univariate and multivariate analyses for risk factors for severe *Strongyloides stercoralis* infection.

Variable	Cases	Controls	Univariate analysis		Multivariate analysis	
	(n = 46)	(n = 92)	OR (95% CI)	*P*	OR (95% CI)	*P*
**Age (years), mean (± SD)**	48.48 (±13.77)	46.25 (±11.79)	1.01 (0.98–1.04)	0.32		
**Male, n (%)**	28 (60.9)	61 (66.3)	0.8 (0.4–1.6)	0.57		
**Race, n (%)**						
White	24 (53.3)^1^	48 (53.9)^2^	1			
Mixed	10 (22.2)^1^	27 (30.3)^2^	0.7 (0.3–1.8)	0.4		
Black	11 (24.4)^1^	14 (15.7)^2^	1.6 (0.6–4.0)			
**Months on dialysis before transplantation, median (IQR)**	42.5 (27–91)	35 (17–60)	1.01 (1.00–1.02)	0.07		
**Deceased donor, n (%)**	40 (87)	44 (47.8)	7.3 (2.8–18.8)	< 0.001	6.16 (2.05–18.5)	0.001
**Prophylaxis, n (%)**	28 (60.9)	37 (40.2)	2.3 (1.1–4.8)	0.03		
**Induction therapy, n (%)**	18 (39.1)	18 (19.6)	2.6 (1.2–5.8)	0.02		
**Delayed graft function, n (%)**	30 (65.2)	29 (31.5)	4.1 (1.9–8.6)	< 0.001		
**Graft rejection, n (%)**	13 (28.3)	13 (14.1)	2.4 (1.0–5.7)	0.06		
**Immunosuppressants, n (%)**						
Tacrolimus	34 (73.9)	63 (68.5)	1.3 (0.6–2.9)	0.56		
Cyclosporine	0 (0)	19 (20.7)	–	< 0.001	0.02 (0.0005–1.03)	0.05
Mycophenolate sodium/mofetil	32 (69.6)	66 (71.7)	0.9 (0.4–1.9)	0.8		
Azathioprine	3 (6.5)	13 (14.1)	0.4 (0.1–1.6)	0.26		
Everolimus/Sirolimus	12 (26.1)	15 (16.3)	1.8 (0.8–4.3)	0.18		
**Cumulative corticosteroid dose (mg/kg), median (IQR)**	73.32 (40.93–157.46)	65.23 (32.05–155.28)	1.001 (0.998–1.003)	0.53	1.005 (1.001–1.009)	0.008
**Previous cytomegalovirus infection, n (%)**	15 (32.6)	16 (17.4)	2.3 (1.0–5.2)	0.053		
**Other previous viral infection, n (%)**	6 (13)	8 (8.7)	1.6 (0.5–4.8)	0.5		
**Previous mycobacterial infection, n (%)**	1 (2.2)	1 (1.1)	2.0 (0.1–33.1)	1.00		
**Previous fungal infection, n (%)**	6 (13)	3 (3.3)	4.4 (1.1–18.7)	0.06	5.63 (0.98–32.19)	0.05
**Previous bacterial infection, n (%)**	22 (47.8)	17 (18.5)	4.0 (1.8–8.8)	0.001	3.04 (1.2–7.5)	0.015
**Previous protozoan infection, n (%)**	0 (0)	1 (1.1)	–	1		

^1^ n = 45

^2^ n = 89.

**Fig 2:**
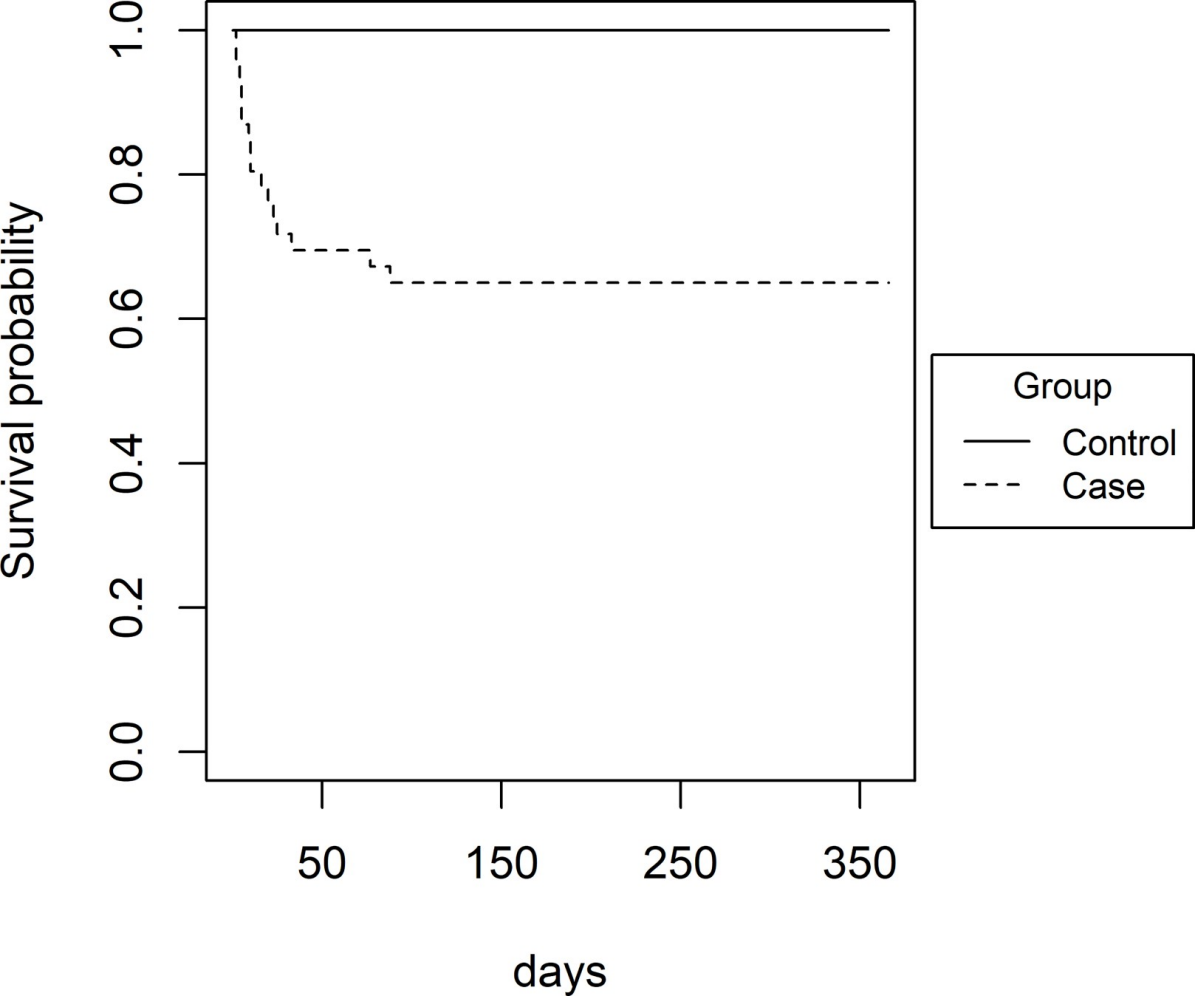
One-year survival curves for patients with and without a diagnosis of severe *Strongyloides stercoralis* infection after solid organ transplantation (cases and controls, respectively).

The estimated probability of survival was significantly lower for the cases than for the controls (*P* < 0.001; Fig 2). Table 6 presents the univariate and multivariate analysis of risk factors associated with 30-day all-cause mortality among the 43 treated cases of severe *S*. *stercoralis* infection. Respiratory failure was independently associated with 30-day mortality (OR = 98.33, 95% CI = 4.46–2169.77, *P* = 0.004), as was gram-negative bacteremia (OR = 413, 95% CI = 4.83–35316.61, *P* = 0.008). In the univariate analysis, the use of any kind of prophylaxis against *S*. *stercoralis* infection was found to be a protective factor (OR = 0.2; 95% CI = 0.03–0.76; *P* = 0.024), although it did not remain significant in the multivariate analysis.

**Table 6:** Univariate and multivariate analysis of risk factors for 30-day mortality among treated patients with severe *Strongyloides stercoralis* infection.

	Survivors	Nonsurvivors	Univariate analysis		Multivariate analysis	
	(n = 33)	(n = 10)	OR (95% CI)	*P*	OR (95% CI)	*P*
**Age (years), mean (±SD)**	47.24 (±14.83)	50.7 (±11.6)	1.02 (0.97–1.07)	0.50		
**Male, n (%)**	18 (54.5)	7 (70)	1.9 (0.4–8.9)	0.48		
**Race, n (%)**				0.31		
White	19 (57.6)	4 (44.4)^1^	1			
Mixed	6 (18.2)	4 (44.4)^1^	3.17 (0.6–16.7)			
Brown	8 (24.2)	1 (11.1)^1^	0.6 (0.06–6.2)			
**Prophylaxis, n (%)**	24 (72.7)	3 (30)	0.2 (0.03–0.76)	0.024		
**Days from transplantation to infection, median (IQR)**	173 (88–1518)	78 (60–120)	3.8 (0.7–20.5)^2^	0.01		
**Days from admission to diagnosis, median (IQR)**	6 (3–11)	7.5 (5–22)	1.09 (1–1.2)	0.3		
**Deceased donor, n (%)**	27 (81.8)	10 (100)		0.31		
**Induction therapy, n (%)**	20 (60.6)	7 (70)	1.5 (0.33–6.95)	0.72		
**Mean daily dose of prednisone (mg), median (IQR)**	19.95 (9.38–43.7)	38.2 (25.1–60.7)	1.03 (1.00–1.06)	0.02		
**Eosinophilia, n (%)**	16 (48.5)	4 (40)	0.71 (0.17–2.98)	0.73		
**Septic shock, n (%)**	4 (12.1)	4 (40)	4.83 (0.94–24.95)	0.07		
**Gram-negative bacteremia, n (%)**	0	3 (30)		0.01	413.00 (4.83–35316.61)	0.008
**Respiratory failure, n (%)**	4 (12.1)	7 (70)	16.92 (3.06–93.48)	0.001	98.33 (4.46–2169.77)	0.004
**Significant graft dysfunction, n (%)**	2 (6.1)	5 (50)	15.5 (2.34–102.85)	0.004		
**Treatment, n (%)**						
Monotherapy	15 (45.4)	3 (30)	1	0.48		
Combination therapy	18 (54.5)	7 (70)	1.94 (0.43–8.86)			
Ivermectin (as monotherapy or in combination)	28 (84.85)	7 (70)	0.42 (0.08–2.18)	0.36		

^1^ n = 9

^2^ reference category ≤ 180 days.

## Discussion

Strongyloidiasis is endemic in low- and middle-income countries, potentially causing severe morbidity and death in organ transplant recipients [15,16]. To our knowledge, this is the first multicenter study to assess risk factors for severe *S*. *stercoralis* infections and the predictors of mortality associated with this complication in kidney transplant recipients.

There is no strict criterion for the diagnosis of severe *S*. *stercoralis* infection. Some authors use the term hyperinfection syndrome, meaning enhancement of symptoms related to an increased worm burden attributed to an accelerated autoinfection cycle. Other criteria include isolation of worms outside of the organs in which they usually complete their life cycle. In the present study, we opted to focus on clinical symptoms rather than identifying any specific form of the disease. Clinical and parasitological diagnostic terminology and commonly adopted study models are not concerned with identifying such forms. Grouping cases within the severe definition facilitates data collection and communication, as well as facilitating the differentiation and exclusion of mild infections [5,7,12]. Irrespective of the diagnostic terminology, the diagnosis of severe *S*. *stercoralis* infection is frequently delayed due to the lack of specificity of clinical symptoms [17,18]. In the present study, we have shown interesting elements to increase the level of suspicion. Similar to what has been reported previously [22], clinical manifestations that were suggestive of the diagnosis were mainly gastrointestinal complaints, and the level of suspicion was increased when gastrointestinal symptoms were accompanied by respiratory symptoms. The absence of fever and eosinophilia should not rule out the diagnosis.

Previous studies of parasitic infections in solid organ transplant recipients have focused on severe disease occurring early after transplantation, usually as a result of donor-transmitted infection or early reactivation of intestinal worms [19–21]. In our study, infection was diagnosed within the first three months after transplantation in 36.9% of the cases, whereas it was diagnosed more than one year after transplantation in 28.3%. The cases occurring later were probably due to persistent community exposure after hospital discharge, which suggests *de novo* infection, reinfection, or reactivation of latent infection. Therefore, it should be borne in mind that severe *S*. *stercoralis* infection can occur long after transplantation, which could have an impact on prophylaxis practices.

Mortality associated with *S*. *stercoralis* infection has declined in recent decades, from rates as high as 50% to rates as low as 28% [19,23], similar to that observed in the present study. That decrease can probably be explained by greater awareness on the part of clinicians, attributable to clinical experience as well as to increased numbers of published articles on the topic, both of which have facilitated the identification of severe *S*. *stercoralis* infection and have improved its management. Despite that decline, crude mortality rates are still unacceptably high. Clinical complications related to the progression of severe *S*. *stercoralis* infection can worsen the prognosis, as was evidenced in our sample. We found gram-negative bacteremia and respiratory failure to be independent predictors of mortality, as has been shown in previous studies [7,24]. Certainly, delayed treatment as a result of late diagnosis can lead to clinical complications. Among our cases, the median time from hospital admission to diagnosis was relatively short and did not differ significantly between the survivors and nonsurvivors (6 days *vs*. 7.5 days). However, we were unable to collect data on the time from the onset of clinical symptoms to diagnosis, given that many mild manifestations might have been present before hospital admission.

Coinfection with CMV occurred in approximately one quarter of our cases, which is in keeping with the findings of previous studies [9,25,26]. It is possible that the patients were so immunosuppressed that a latent CMV infection was reactivated. Alternatively, CMV infection could have been reactivated due to the intense pro-inflammatory state generated by severe *S*. *stercoralis* infection, similarly to what has been reported in critically ill patients [27,28]. Because symptoms may overlap, our findings suggest that active surveillance for CMV infection in the course of severe *S*. *stercoralis* infection should be mandatory.

In the present study, we have identified specific risk factors for severe *S*. *stercoralis* infection. Recipients of deceased donor transplants are submitted to more intense immunosuppression due to a higher risk of graft rejection. The association between a high cumulative corticosteroid dose and such infection is well documented. Corticosteroids reduce the total count of eosinophils, which play a relevant role as antigen-presenting cells in the immune response to helminths. In addition, corticosteroids may play a role in promoting the transition of *S*. *stercoralis* larvae from the rhabditiform stage to the filariform stage, thus increasing the rate of intestinal autoinfection [15,29–33]. On the basis of our data, a cumulative dose of 5 g of prednisone per 70 kg of body weight could be used as a cutoff value for risk estimation. On interesting finding of the present study is that previous bacterial infections were independently associated with severe *S*. *stercoralis* infection. It is possible that previous antibiotic use alters the gut microbiome and increases the rate of local *S*. *stercoralis* replication and reinfection, although this hypothesis should be confirmed in specific studies. Alternatively, previous bacterial infections could indicate a higher degree of immunosuppression.

It has been proposed that prophylaxis against *S*. *stercoralis* infection should be used in order to decrease the risk of severe *S*. *stercoralis* infection, because there is some evidence that it is protective, although its benefit has not been tested in clinical trials [7,19,34–36]. In the present study, we found no protective effect of such prophylaxis, even regarding infection occurring within the first three months after transplantation. That might be due to the use of prophylactic agents other than ivermectin, which could have affected our results, given that earlier studies and a recent meta-analysis have shown the superiority of ivermectin over albendazole in *S*. *stercoralis* eradication in stool specimens [37]. However, our analysis of risk factors for mortality showed a protective role of prophylaxis in the univariate model. Although that could be explained by a lower parasite burden at the time of infection, it should be explored in larger studies. In addition, despite the fact that less effective drugs were employed, mortality was lower among the cases in which prophylaxis was administered, probably because it avoided early infection. Prophylaxis is usually employed at the time of transplantation to prevent donor-transmitted infection. Here, we have shown that, in Brazil, a country with high rates of community transmission of *S*. *stercoralis*, a considerable proportion of kidney transplant recipients develop severe *S*. *stercoralis* infection late after transplantation, suggesting either that prophylaxis should be repeated periodically after transplantation or appropriate surveillance should be conducted for the identification and treatment of infected patients [38]. Although we found no beneficial effect of prophylaxis, its administration early after transplantation, preferably with ivermectin, is a valid recommendation, given that anthelmintic treatment is safe and might reduce mortality.

We found that severe *S*. *stercoralis* infection occurring within the first three months after transplantation has a more critical clinical course, with a significantly higher rate of respiratory failure and a significantly higher 30-day mortality rate. This three-month cutoff point was chosen arbitrarily in an attempt to differentiate disease due to donor-transmitted infection or early reactivation of latent infection from that due to late reactivation or community acquisition, although an overlap cannot be ruled out. There are two possible explanations for the more critical clinical course. First, donor-transmitted infection might occur in the context of a higher parasite burden, *S*. *stercoralis* larvae seeding directly into the bloodstream. The second possibility is that the disease manifests in a period of more intense immunosuppression, sufficient to increase the larval burden.

Our study has some limitations. First, the retrospective nature precluded the real-time assessment of infection/disease. As a result, the true incidence of severe *S*. *stercoralis* infection might have been underestimated. Second, we were unable to collect data on the human T-lymphotropic virus (HTLV) infection status of the recipients, a variable that has been shown to affect the clinical course of *S*. *stercoralis* infection [39,40]. Such information is not systematically collected for transplant recipients in Brazil. Although the actual prevalence of HTLV infection after kidney transplantation is unknown, many studies have found low rates of HTLV seropositivity in transplant recipients [39,40]. Although there have been some reports of myelopathy after donor-transmitted infection, it seems that the clinical course of infection in recipients testing positive for HTLV prior to transplantation is benign even under immunosuppression [39,40]. In addition, there have been no reports of an association between HTLV and strongyloidiasis in transplant recipients, although most studies of the topic have been conducted in countries with a low prevalence of strongyloidiasis [39,40]. Finally, we had no access to data on donor or recipient screening for *S*. *stercoralis* infection before transplantation. Given that *S*. *stercoralis* infection rates in Brazil are reported to be as high as 29% [2] and that diagnostic tests (serology and stool evaluation) are often unavailable or have limitations, as well as that anthelmintic drugs are affordable, have low toxicity, and are widely available, transplant centers in Brazil usually take it for granted that infection is present before transplantation. Therefore, the current practice is either to provide prophylaxis for recipients or to treat apparent infection. Screening for strongyloidiasis in donors and recipients is not an easy task. Antibody detection methods, stool evaluation, and more recently polymerase chain reaction techniques are available for diagnosis. Major limitations are the low specificity of antibody detection methods, the low sensitivity of stool evaluation, and the limited experience with, as well as the lack of methodological standardization of polymerase chain reaction. Due to time constraints and the limited availability of laboratory screening tests, the donor is rarely screened. One recent study of 58 organ procurement organizations in the United States showed that only 10% screened donors for strongyloidiasis [41]. However, a beneficial effect of screening was reported in a single-center study conducted by Camargo et al. [42], who reported that 14% of organs obtained from deceased donors were seropositive for *S*. *stercoralis*. In that study, all recipients received prophylaxis with ivermectin and remained free from strongyloidiasis throughout the study follow-up period [42]. The screening of transplant recipients could reduce the unnecessary use of ivermectin. Although antibody detection methods lack specificity for *S*. *stercoralis* infection (a major concern in low- and middle-income countries where other helminth infections are prevalent), such tests could be useful because of their high negative predictive value [43]. In our study, it was difficult to confirm donor-transmitted infection because there was no pre-transplant screening. However, because donor-transmitted infection usually leads to early occurrence of disease, we think the great majority of such infections are represented in our group of patients in whom severe *S*. *stercoralis* infection occurred within the first three months after transplantation.

### Conclusions

We have shown that severe *S*. *stercoralis* infection is still a relevant issue in kidney transplantation, with significant morbidity and mortality, especially when it occurs early after transplantation. Some of our findings could be translated to clinical practice to improve outcomes of severe *S*. *stercoralis* infection. For example, the early diagnosis of severe *S*. *stercoralis* infection can be improved on the basis of clinical suspicion. Abdominal distension with epigastric pain in the absence of a surgical condition, especially if accompanied by respiratory symptoms, should raise the suspicion of severe *S*. *stercoralis* infection. Fever and eosinophilia may not always be present, and their absence should not be used to rule out severe *S*. *stercoralis* infection. In addition, given the limitations of current diagnostic methods, invasive methods should be employed if necessary in order to make a proper diagnosis. Furthermore, after the establishment of severe *S*. *stercoralis* infection, active surveillance for infection with CMV and gram-negative bacilli should be performed and empirical antibiotic coverage for gram-negative bacilli could be employed. Moreover, knowledge of risk factors such as previous bacterial infections, high cumulative corticosteroid use (more than 5g for a 70-kg patient) and receiving a graft from a deceased donor could be used to strengthen the suspicion of severe *S*. *stercoralis* infection. In endemic regions, severe *S*. *stercoralis* infection can occur late after transplantation. Diagnostic and prophylactic strategies should take that into account. The risk of *de novo* infection warrants either periodic prophylaxis or diagnostic screening. Finally, *S*. *stercoralis* prophylaxis may have an impact on mortality, probably by delaying disease occurrence, and should be employed in patients deemed to be at risk for infection.

## Supporting information

S1 Checklist STROBE checklist. (DOC)
