# Correction: Severe *Strongyloides stercoralis* infection in kidney transplant recipients: A multicenter case-control study

**DOI:** 10.1371/journal.pntd.0009342

**Published:** 2021-04-12

**Authors:** The PLOS Neglected Tropical Diseases Staff

The third author’s name is spelled incorrectly. The publisher apologizes for the error. The correct name is: Guilherme Santoro-Lopes. The correct citation is: Miglioli-Galvão L, Pestana JOM, Santoro-Lopes G, Torres Gonçalves R, Requião Moura LR, Pacheco Silva Á, et al. (2020) Severe *Strongyloides stercoralis* infection in kidney transplant recipients: A multicenter case-control study. PLoS Negl Trop Dis 14(1): e0007998. https://doi.org/10.1371/journal.pntd.0007998
