# Supplementary material for: Severe Strongyloides stercoralis infection in kidney transplant recipients: A multicenter case-control study
Source: PLoS Negl Trop Dis. 2020 Jan 31;14(1):e0007998. doi: 10.1371/journal.pntd.0007998 (PMC7015428; doi:10.1371/journal.pntd.0007998)
Supplement: S1 Checklist — (DOC) [file pntd.0007998.s001.doc]

STROBE Statement—Checklist of items that should be included in reports of ***case-control studies***

|  | Item No | Recommendation |
| --- | --- | --- |
| **Title and abstract** | 1 | (*a*) Indicate the study’s design with a commonly used term in the title or the abstract – paragraph 1 |
|  |  | (*b*) Provide in the abstract an informative and balanced summary of what was done and what was found – paragraph 2 |
| Introduction | | |
| Background/rationale | 2 | Explain the scientific background and rationale for the investigation being reported – paragraph 77 |
| Objectives | 3 | State specific objectives, including any prespecified hypotheses – paragraph 97 |
| Methods | | |
| Study design | 4 | Present key elements of study design early in the paper – paragraph 109 |
| Setting | 5 | Describe the setting, locations, and relevant dates, including periods of recruitment, exposure, follow-up, and data collection – paragraph 109 |
| Participants | 6 | (*a*) Give the eligibility criteria, and the sources and methods of case ascertainment and control selection. Give the rationale for the choice of cases and controls – paragraph 114 |
|  |  | (*b*) For matched studies, give matching criteria and the number of controls per case – paragraph 126 |
| Variables | 7 | Clearly define all outcomes, exposures, predictors, potential confounders, and effect modifiers. Give diagnostic criteria, if applicable – no applicable |
| Data sources/ measurement | 8* | For each variable of interest, give sources of data and details of methods of assessment (measurement). Describe comparability of assessment methods if there is more than one group – paragraph 138 |
| Bias | 9 | Describe any efforts to address potential sources of bias – no applicable |
| Study size | 10 | Explain how the study size was arrived at – no applicable |
| Quantitative variables | 11 | Explain how quantitative variables were handled in the analyses. If applicable, describe which groupings were chosen and why – paragraph 167 |
| Statistical methods | 12 | (*a*) Describe all statistical methods, including those used to control for confounding – paragraph 167 |
|  |  | (*b*) Describe any methods used to examine subgroups and interactions - paragraph 167 |
|  |  | (*c*) Explain how missing data were addressed- paragraph 167 |
|  |  | (*d*) If applicable, explain how matching of cases and controls was addressed - paragraph 167 |
|  |  | (*e*) Describe any sensitivity analyses – no applicable |
| Results | | |
| Participants | 13* | (a) Report numbers of individuals at each stage of study—eg numbers potentially eligible, examined for eligibility, confirmed eligible, included in the study, completing follow-up, and analysed - paragraph 184 |
|  |  | (b) Give reasons for non-participation at each stage - paragraph 184 |
|  |  | (c) Consider use of a flow diagram – no applicable |
| Descriptive data | 14* | (a) Give characteristics of study participants (eg demographic, clinical, social) and information on exposures and potential confounders - paragraph 201 |
|  |  | (b) Indicate number of participants with missing data for each variable of interest – no applicable |
| Outcome data | 15* | Report numbers in each exposure category, or summary measures of exposure – paragraph 184 |
| Main results | 16 | (*a*) Give unadjusted estimates and, if applicable, confounder-adjusted estimates and their precision (eg, 95% confidence interval). Make clear which confounders were adjusted for and why they were included - paragraph 201 |
|  |  | (*b*) Report category boundaries when continuous variables were categorized - paragraph 201 |
|  |  | (*c*) If relevant, consider translating estimates of relative risk into absolute risk for a meaningful time period - paragraph 201 |

| Other analyses | 17 | Report other analyses done—eg analyses of subgroups and interactions, and sensitivity analyses – no applicable |
| --- | --- | --- |
| Discussion | | |
| Key results | 18 | Summarise key results with reference to study objectives - paragraph 333 |
| Limitations | 19 | Discuss limitations of the study, taking into account sources of potential bias or imprecision. Discuss both direction and magnitude of any potential bias – paragraph 439 |
| Interpretation | 20 | Give a cautious overall interpretation of results considering objectives, limitations, multiplicity of analyses, results from similar studies, and other relevant evidence - paragraph 484 |
| Generalisability | 21 | Discuss the generalisability (external validity) of the study results – no applicable |
| Other information | | |
| Funding | 22 | Give the source of funding and the role of the funders for the present study and, if applicable, for the original study on which the present article is based - no applicable |

*Give information separately for cases and controls.

**Note:** An Explanation and Elaboration article discusses each checklist item and gives methodological background and published examples of transparent reporting. The STROBE checklist is best used in conjunction with this article (freely available on the Web sites of PLoS Medicine at http://www.plosmedicine.org/, Annals of Internal Medicine at http://www.annals.org/, and Epidemiology at http://www.epidem.com/). Information on the STROBE Initiative is available at http://www.strobe-statement.org.
